# Research on Innovative Shale Gas Exploitation and Utilization System Based on CO_2_ Integrated with Displacement, Power Generation and Refrigeration

**DOI:** 10.3390/e27121199

**Published:** 2025-11-26

**Authors:** Shengya Hou, Feifei Jiao, Fengyuan Zhang, Qiguo Yang

**Affiliations:** 1 School of Energy and Power Engineering, University of Shanghai for Science and Technology, Shanghai 200093, China; yangqg@usst.edu.cn; 2 School of Information Science and Technology, ShanghaiTech University, Shanghai 201210, China; jiaoff@shanghaitech.edu.cn; 3 Department of Chemical Engineering, University College London, Torrington Place, London WC1E 7JE, UK

**Keywords:** shale gas exploitation, supercritical CO_2_ power cycle, transcritical CO_2_ refrigeration cycle, gas turbine power generation, integrated system

## Abstract

This paper presents a novel, integrated supercritical CO_2_ system for shale gas development, comprising a supercritical CO_2_ shale gas extraction system, a gas turbine system, a supercritical CO_2_ power generation system, and a transcritical CO_2_ refrigeration system. A comprehensive thermodynamic and economic analysis is conducted for this integrated energy development system. To enhance system performance across multiple dimensions, three objective functions are proposed for optimization: exergy efficiency, levelized energy cost (LEC), and heat transfer area per unit power output (APR). First, the effects of key operating parameters—including the gas turbine pressure ratio, gas turbine inlet temperature, supercritical CO_2_ pressure ratio, the temperature difference between flue gas and the supercritical CO_2_ top cycle, and the temperature difference between flue gas and the supercritical CO_2_ bottom cycle—on system performance were analyzed through parametric studies. Next, the optimal system parameters were determined using a multi-objective optimization method based on a genetic algorithm. The optimization results reveal that, when exergy efficiency and LEC are used as dual-objective functions, the system achieves an optimal exergy efficiency of 60.5% and an LEC of 6.3 cents/(kW·h). Furthermore, when exergy efficiency, APR, and LEC are considered as objective functions, the system attains an optimal exergy efficiency of 59.5%, an APR of 0.21 m^2^/kW, and an LEC of 6.3 cents/(kW·h). The compound shale gas development system proposed in this paper demonstrates excellent economic viability, environmental sustainability and operational efficiency. The research outcomes offer an innovative solution for the development of shale gas and contribute to the advancement of research on new energy systems.

## 1. Introduction

As a relatively clean fossil fuel, shale gas can act as a crucial transitional energy source in the process of transitioning to renewable energy, which contributes to the reduction of carbon emissions [1,2,3]. Currently, hydraulic fracturing is the primary method for shale gas extraction. However, hydraulic fracturing gives rise to a series of issues, including water resource consumption, water pollution risks, geological hazards, and seismic risks [4,5,6,7,8].

In contrast to conventional hydraulic fracturing techniques, supercritical carbon dioxide (S-CO_2_) exhibits distinct advantages attributable to its unique characteristics. It combines the high diffusivity and low viscosity typical of gases with the high-density characteristic of liquids. Moreover, it has an extremely low surface tension, enabling it to penetrate into minuscule pores. These characteristics, in conjunction with its favorable thermodynamic and kinetic properties, lead to a substantially higher recovery efficiency of supercritical carbon dioxide compared to that of traditional fracturing fluids [9]. Shen Wei’s study reveals that supercritical carbon dioxide not only decreases the fracturing pressure of shale formations but also facilitates the propagation of fractures, giving rise to complex fracture networks and enhancing reservoir permeability [10]. In the course of shale gas exploitation, a fraction of the injected carbon dioxide can be securely sequestered within the shale reservoir, thus contributing to the reduction of carbon emissions [11]. Kuang N. put forward that the ability of supercritical CO_2_ to enhance shale gas recovery can be attributed to mechanisms such as its greater competitive adsorption capacity, the increase in CH_4_ partial pressure, and the acceleration of CH_4_ diffusion [12].

Prior to the injection of carbon dioxide, a refrigeration system, typically compression refrigeration [13], is requisite to maintain carbon dioxide in a liquid state. Nevertheless, the compression refrigeration approach may give rise to severe environmental concerns. Consequently, a transcritical CO_2_ refrigeration system can be contemplated as an alternative solution. As an environmentally benign refrigerant, carbon dioxide exhibits an ozone depletion potential (ODP) of zero and a global warming potential of 1. Moreover, in contrast to conventional refrigerants, carbon dioxide is non-toxic and non-flammable, rendering it a safer option for the environment [14]. Carbon footprint analysis indicates that, when compared to a system utilizing the traditional refrigerant R404A, an improved system employing CO_2_ as a refrigerant can lead to a 17.95% reduction in carbon emissions [15]. With its high density and low viscosity, CO_2_ as a refrigerant mitigates flow losses and enhances heat transfer efficiency, thereby attaining rapid and efficient refrigeration performance [16].

To improve the energy utilization efficiency of shale gas and reduce transportation costs, on-site power generation using shale gas-fueled gas turbines can be implemented. Given that the exhaust gases from gas turbines remain at elevated temperatures, system efficiency can be further enhanced through waste heat recovery technologies. Conventional waste heat recovery systems typically employ the steam Rankine cycle; however, their potential for efficiency improvement is inherently limited. Therefore, the supercritical carbon dioxide (S-CO_2_) Brayton cycle has been proposed as a more effective alternative for waste heat recovery. The S-CO_2_ Brayton cycle exhibits notable advantages, including high compactness and superior thermal efficiency within the temperature range of 450–750 °C, making it particularly suitable for recovering waste heat from gas turbine exhaust [17]. CAO conducted an optimization study on the application of the supercritical CO_2_ cycle in gas turbine waste heat recovery. The results demonstrated that the optimal technical–economic configuration achieves a levelized cost of electricity of 0.0420 $/kWh and a net power output of 19.701 MW [18]. Chen systematically compared various configurations of supercritical CO_2_ cycles for gas turbine waste heat recovery and identified the intercooled cycle as the most optimal. This configuration achieves a thermal efficiency of 0.406 and a cost rate of 2.724 $/GJ, respectively [19].

In light of the above, existing research is lacking in a comprehensive energy system for shale gas extraction using carbon dioxide. Thus, this study puts forward a novel integrated energy system that encompasses storage, extraction, and utilization processes. Carbon dioxide serves as the sole working fluid throughout all stages of the process, enabling its effective reuse and sequestration, thereby achieving high environmental performance and offering a promising pathway for sustainable shale gas development. Furthermore, a multi-objective optimization of the integrated energy system is conducted. By enhancing the performance of the energy system from multiple perspectives, this optimization aims to resolve issues encountered during the shale gas utilization process. This effort is conducive to accelerating the development of the technology of using supercritical carbon dioxide for shale gas extraction.

## 2. Cycle Description and Assumptions

**Figure 1:**
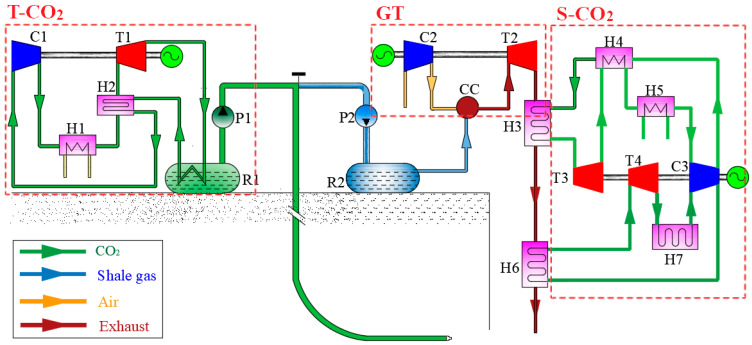
Schematic diagram of the proposed system.

To address energy and environmental challenges associated with the greenhouse effect, the characteristics of shale gas exploitation are taken into full consideration. Supercritical CO_2_ is used to exploit shale gas, and the extracted shale gas is directly burned and generated by gas turbine. The expanded flue gas still has a high temperature and can be used as the heat source of supercritical CO_2_ power cycle. In the process of exploiting shale gas with supercritical CO_2_, the temperature of CO_2_ supplied should be maintained at about −20 °C, and the trans-critical CO_2_ refrigeration system can meet the requirements. Therefore, the proposed integrated system comprises a supercritical CO_2_ mining subsystem, a gas turbine subsystem, a supercritical CO_2_ power generation subsystem, and a transcritical CO_2_ refrigeration subsystem, as illustrated in Figure 1.

The drilling system for shale gas extraction using supercritical CO_2_ primarily comprises a CO_2_ storage tank (R1), a CO_2_ delivery pump (P1), and a drilling pipeline system. The CO_2_ in the storage tank is maintained at a pressure of 4 MPa and a temperature range of −20 to 0 °C. A transcritical CO_2_ refrigeration cycle is employed to ensure the CO_2_ remains in a liquid state. The liquid CO_2_ is pumped from the storage tank to the coiled tubing via the delivery pump. At the bottom of the coiled tubing, a directional device, whipstock, and a flexible rod assembly are installed. The flexible rod is designed to bend within a specific range, transitioning from vertical to horizontal at the bottom of the wellbore. A supercritical CO_2_ injection drill bit is mounted at the end of the assembly to facilitate the drilling process.

The transcritical CO_2_ refrigeration cycle is employed to maintain the temperature of CO_2_ in storage tank (R1). This cycle uses CO_2_ as the working medium, ensuring its environmental friendliness. The transcritical refrigeration system comprises a CO_2_ storage tank (R1), a compressor (C1), an expander (T1), an air cooler (H1), and a regenerator (H2). In this system, CO_2_ is compressed in the compressor (C1) to a pressure exceeding the critical pressure. The high-pressure CO_2_ is then cooled in the air cooler (H1) and further cooled by the regenerator (H2), thereby increasing the refrigeration coefficient. After expansion through the expander (T1), the cooled CO_2_ absorbs heat from the CO_2_ storage tank (R1) and the regenerator (H2) before re-entering the compressor (C1) for compression, thus completing one cycle. A key feature of this cycle is that the evaporation process occurs in the subcritical region, while the condensation process takes place in the supercritical region. The energy required to drive the transcritical CO_2_ compressor is supplied by electricity generated from a gas turbine.

The extracted shale gas can be utilized directly at the tunnel outlet and delivered to the combustion chamber (CC) of the gas turbine system via the delivery pump (P2). Compressed air from the compressor (C2) enters the combustion chamber, where it mixes with shale gas for combustion, generating high-temperature flue gas. This flue gas expands in the turbine (T2) to produce electricity. A portion of the electricity generated by the gas turbine is used to power the compressor (C1) in the transcritical CO_2_ refrigeration cycle, while the remaining electricity is transmitted to the grid through high-voltage transmission lines.

The flue gas at the outlet of the gas turbine retains a high temperature, making it an ideal heat source for the S-CO_2_ Brayton cycle. This S-CO_2_ power system consists of an expander (T3), a compressor (C3), a cooler (H5), and a heat absorber (H3). To recover residual heat from the CO_2_ at the expander outlet, a regenerator (H4) is included in the design. After being compressed in the compressor (C3), CO_2_ flows through the regenerator and heat absorber (H3) to absorb heat. The high-temperature, high-pressure CO_2_ then enters the expander (T3), where it expands to generate electricity. The expanded CO_2_ subsequently passes through the regenerator and cooler to release heat before returning to the compressor, completing the cycle. Since the flue gas temperature at the outlet of the heat absorber (H3) remains high, a secondary supercritical CO_2_ cycle is integrated as the bottom cycle. This bottom cycle includes an expansion machine (T4), a compressor (C3), a cooler (H7), and a heat absorber (H3). Notably, the compressor (C3) is shared between the top and bottom supercritical CO_2_ cycles, optimizing system integration and efficiency.

## 3. Mathematical Modeling

To analyze and optimize the performance of the integrated shale gas development and utilization system, a computational model was developed. This model integrates a thermodynamic model, a thermo-economic model, and a system compactness assessment model. The evaluation metrics employed include exergy efficiency, levelized cost of energy (LCE), and area per unit power output (APR) of the heat exchanger.

The assumptions of the model are as follows [20,21]:

(1) The system maintains a steady-state operating condition.
(2) The temperature and pressure of the environmental conditions are 25 °C and 1.013 bar, respectively.
(3) The variations in potential energy and kinetic energy are negligible.

### 3.1. Thermodynamic Analysis Model

#### 3.1.1. Heat Exchanger Model

Under supercritical conditions, carbon dioxide exhibits gas-like behavior, which gives rise to relatively inferior heat transfer properties. Consequently, a highly efficient heat-exchange device is indispensable. Printed circuit heat exchangers (PCHE) feature a high surface-area-to-volume ratio and possess excellent resistance to high temperatures and pressures. Therefore, in this research, the PCHE has been identified as the optimal heat-exchange device.

In the system, significant variations exist in the thermophysical properties of the hot and cold fluids within the printed circuit heat exchanger. Particularly near the pseudocritical temperature of carbon dioxide, the specific heat at constant pressure exhibits a sharp peak, leading to pronounced nonlinearity in thermal behavior. Applying the logarithmic mean temperature difference (LMTD) method over the entire heat transfer process would result in considerable inaccuracies due to this property variation. Therefore, in this study, the heat transfer calculation is performed by dividing the printed circuit heat exchanger into *n* discrete segments with equal incremental heat transfer. In the calculation process, the value of *n* is set to 50. The local logarithmic mean temperature difference for both hot and cold fluids is computed in each segment.

Based on the foregoing analysis, the following relationships can be established:

(1) $$\Delta Q_{k}=\frac{Q}{n}.$$

(2) $$\Delta Q_{k}=U\Delta A_{k}\Delta T_{k}.$$

(3) $$\Delta Q_{k}={\dot{m}}_{h,k}(h_{hk.i}-h_{hk,o})={\dot{m}}_{c,k}(h_{ck,o}-h_{ck,i}).$$

(4) $$\sum \Delta A_{k}=A.$$

The efficiency of each unit is defined as follows:

(5) $$\varepsilon =\frac{\Delta Q_{k}}{\Delta Q_{k,\max}}.$$

In the formula, $\Delta Q_{k,\max}$ represents the ideal maximum thermal power, defined as:

(6) $$\Delta Q_{k,\max}=C_{k,\min}(T_{hk,i}-T_{ck,i}).$$

where $C_{k,\min}$ is the smaller heat capacity of the two fluids:

(7) $$C_{k,\min}=min\left({\dot{m}}_{h}c_{Ph,k},{\dot{m}}_{c}c_{Pc,k}\right).$$

The effectiveness of the heat exchanger unit can be alternatively expressed as

(8) $$NTU_{k}=\frac{U\Delta A_{k}}{C_{k,\min}}.$$

(9) $$\varepsilon_{k}=\frac{1-\exp\left(-NTU_{k}\left(1-R_{k}\right)\right)}{1-R_{k}\exp\left(-NTU_{k}\left(1-R_{k}\right)\right)}.$$

In the formula, $R_{k}$ represents the ratio of the smaller heat capacity to the larger heat capacity of the two fluids, defined as:

(10) $$R_{k}=\frac{C_{k,\min}}{\max\left({\dot{m}}_{h}c_{ph,k},{\dot{m}}_{c}c_{pc,k}\right)}.$$

In the design procedure, the outlet temperature of the cold fluid is initially assumed and treated as the iteration variable. This assumption is then used to compute the outlet temperature of the hot fluid in the heat exchanger. The inlet temperature of the cold fluid is subsequently obtained through iterative calculations. The iteration process terminates when the difference between the computed and specified cold fluid inlet temperatures lies within the predefined convergence tolerance.

#### 3.1.2. Turbomachinery Model

Supercritical carbon dioxide expands through the turbine to generate power output, playing a critical role in ensuring the safe, reliable, and efficient operation of the supercritical cycle system. Under ideal conditions, the entropy and specific enthalpy at the turbine outlet can be determined from the following equations:

(11) $$s_{t,e^{\prime }}=s_{t,i}$$

(12) $$h_{t,e^{\prime }}=h(s_{t,e^{\prime },}P_{t,e})$$

The actual specific enthalpy at the turbine outlet defined as:

(13) $$h_{t,e}=h_{t,i}-\left(h_{t,i}-h_{t,e^{\prime }}\right)\eta_{t}$$

In the equation, $\eta_{t}$ denotes the isentropic efficiency of the turbine, which is determined by two key dimensionless performance parameters: the specific speed and the specific diameter. These parameters are defined as follows:

(14) $$N_{s}=\frac{w\sqrt{V}}{{\Delta h_{s}}^{\frac{3}{4}}}$$

(15) $$D_{s}=\frac{{D\Delta h_{s}}^{\frac{1}{4}}}{\sqrt{V}}$$

The shaft power of the turbine can be formulated as:

(16) $${\dot{W}}_{t}={\dot{m}}_{t}\left(h_{t,i}-h_{t,e}\right)$$

The cost rate of the supercritical carbon dioxide turbine is expressed as:

(17) $$Z_{t}=\frac{\alpha_{1}{\dot{m}}_{t}}{\eta_{t^{\prime }}-\eta_{t}}\ln\left(PR_{t}\right)\left(1+\exp\left(\alpha_{2}T_{t,i}-K_{t}\right)\right)$$

(18) $$PR_{t}=\frac{P_{t,i}}{P_{t,e}}$$

In the equation, $PR_{t}$ denotes the pressure ratio across the turbine; $\alpha_{1},\alpha_{2},K_{t}$ are coefficients that are determined by the specific type and structural design of the turbine, with values of 479.34, 0.036 and 54.4, respectively.

The exergy loss of fluid within the turbine is defined as:

(19) $${\dot{E}}_{D,t}=\dot{m}(e_{t,i}-e_{t,e})-{\dot{W}}_{t}$$

The thermal efficiency of the supercritical carbon dioxide cycle can be expressed as:

(20) $$\eta_{th}=\frac{{\dot{W}}_{CO_{2}}}{{\dot{Q}}_{CO_{2}}}$$

(21) $$\dot{W}={\dot{W}}_{t}-{\dot{W}}_{c}$$

where ${\dot{Q}}_{CO_{2}}$ represents the heat absorption rate of the system.

### 3.2. Exergy Analysis Model

Exergy analysis is a thermodynamic method based on the second law of thermodynamics, widely employed to evaluate the thermodynamic perfection of energy conversion systems. Unlike thermal efficiency, which only accounts for energy quantity, exergy efficiency provides a more comprehensive assessment by incorporating both energy quality and irreversibility in the conversion process.

The total exergy destruction rate of the S-CO_2_ cycle is given by:

(22) $${\dot{E}}_{D}=\sum_{t=1}^{t=T}{\dot{E}}_{D,H}+{\dot{E}}_{D,t}+{\dot{E}}_{D,c}$$

The total exergy input to the S-CO_2_ cycle is expressed as:

(23) $$\dot{E}=\dot{m}\left(e_{h,i}-e_{h,e}\right)$$

The exergy efficiency of the system is defined as:

(24) $$\eta_{x}=\frac{\dot{W}}{\dot{E}}$$

The coefficient of performance (COP) of the transcritical CO_2_ refrigeration cycle can be defined as:

(25) $$COP=\frac{q_{r}}{W_{c1}}$$

where $q_{r}$ represents the refrigerating capacity of the transcritical carbon dioxide system.

### 3.3. Compactness Model

The compactness of a system is a critical performance parameter. In practical applications, if the system’s volume is excessively large, its practical usability will be severely constrained. Given that heat exchangers typically occupy more than 70% of the total system volume, the heat-transfer area per unit of output power is employed as the objective function to quantitatively evaluate the system’s compactness.

The heat-transfer area per unit of output power is defined as the ratio of the total heat-transfer area required by the system to the output power, and can be expressed as:

(26) $$APR=\frac{\sum_{1}^{F}A_{f}}{\dot{W}}$$

In the equation, F represents the number of heat exchangers within the system, and $A_{f}$ denotes the heat-transfer area of each heat exchanger.

### 3.4. Exergy-Economic Model

Exergy-economic analysis integrates exergy analysis and economic analysis, offering in-depth insights that are unattainable through traditional thermodynamic analysis and economic assessment. In exergy-economic analysis, the exergy cost balance equations and auxiliary equations are applied to each component of the system. By computing the exergy cost of each fluid stream, the exergy cost of the entire system can be minimized [22,23,24].

The exergy cost balance equation for system components is expressed as:

(27) $$\sum {\dot{C}}_{e,k}+{\dot{C}}_{W,k}=\sum {\dot{C}}_{i,k}+{\dot{C}}_{Q,k}+{\dot{Z}}_{k}$$

where ${\dot{Z}}_{k}$ represents the cost rate related to the cost rate attributable to the capital investment, operation, and maintenance expenses.

Among them:

(28) $${\dot{C}}_{e,k}=c_{e,k}{\dot{E}}_{e,k}$$

(29) $${\dot{C}}_{i,k}=c_{i,k}{\dot{E}}_{i,k}$$

(30) $${\dot{C}}_{W,k}=c_{W,k}{\dot{E}}_{W,k}$$

(31) $${\dot{C}}_{Q,k}=c_{Q,k}{\dot{E}}_{Q,k}$$

The cost rate of each component is expressed by:

(32) $${\dot{Z}}_{k}=Z_{k}\cdot CRF\cdot \varphi /(N\times 3600)$$

where *φ* is the correction factor associated with operation and maintenance, with a value of 1.06. *N* represents the annual operating time.

The system investment recovery rate can be defined as:

(33) $$CRF=\frac{i{\left(1+i\right)}^{n}}{{\left(1+i\right)}^{n}-1}$$

where *i* is the real discount rate correction factor and *n* denotes the service life of the system.

The cost rates of product exergy and fuel exergy are expressed as:

(34) $$c_{P,k}=\frac{{\dot{C}}_{P,k}}{{\dot{E}}_{P,k}}$$

(35) $$c_{F,k}=\frac{{\dot{C}}_{F,k}}{{\dot{E}}_{F,k}}$$

The cost rate attributed to exergy destruction is given by:

(36) $${\dot{C}}_{D,k}=c_{F,k}{\dot{E}}_{D,k}$$

Component exergetic efficiency is expressed as:

(37) $$\eta_{ex,k}=\frac{{\dot{E}}_{P,k}}{{\dot{E}}_{in,k}}$$

The exergoeconomic factor, which quantifies the relative significance of investment cost versus exergy loss cost for a given component, is defined as:

(38) $$f_{k}=\frac{{\dot{Z}}_{k}}{{\dot{Z}}_{k}+{\dot{C}}_{D,k}}$$

Finally, the overall objective function of the exergoeconomic model—the cost rate per unit of net output power—is expressed as:

(39) $$\mathrm{LEC}=\frac{\sum_{1}^{n_{k}}{\dot{Z}}_{k}+{\dot{C}}_{fuel}}{\sum_{1}^{n_{p}}{\dot{E}}_{P,i}}$$

where $n_{k}$ and $n_{P}$ denote the total number of product streams and system components, respectively. ${\dot{C}}_{fuel}$ represents the cost rate of fuel combustion, and ${\dot{E}}_{P,i}$ refers to the exergy rate of the *j*th product stream.

### 3.5. Model Validation

To validate the accuracy of the system model employed in this study, a comparative evaluation was conducted between the results obtained from the proposed computational model and relevant published data. Li et al. investigated a carbon dioxide-based cogeneration system for simultaneous cooling and power generation. As the present system incorporates multiple subsystems, separate verification is conducted. Table 1 presents a detailed comparison between the performance indicators computed by the current model and those of the gas turbine and supercritical carbon dioxide cycle system reported in Reference [25]. Table 2 furnishes a detailed comparison between the performance indicators calculated by the current model and those of the transcritical CO_2_ refrigeration cycle system reported in Reference [26]. Among them, the operating input parameters are taken as the values reported in the literature. Upon comparison, it was noted that under identical input conditions, the deviations of the performance parameters of this model from the results in References were all within 5%. This consistency in results serves to confirm the accuracy and reliability of the model developed in this study.

## 4. Results and Analysis

This section presents the results of parameterization and multi-objective optimization of the shale gas development and utilization system. Based on practical application requirements, the optimized performance parameters include exergy efficiency (*η*ₑₓ), levelized energy cost (LEC), and heat transfer area per unit power output (APR). These parameters represent the system’s thermodynamic performance, economic performance, and compactness, respectively.

### 4.1. Parametric Study

Supercritical CO_2_ exhibits a high density comparable to that of a liquid and possesses strong solvation capabilities, making it highly effective in mitigating pollution near wells. Its low-viscosity properties facilitate the formation of micro-fractures that connect natural fractures while preventing clay hydration and expansion. This enhances shale porosity and improves fracture conductivity. Additionally, CO_2_ has an adsorption capacity 4–20 times greater than that of methane, enabling it to effectively displace methane in shale while being sequestered in the reservoir.

Exploiting shale gas with supercritical CO_2_ significantly improves production and recovery efficiency at shale gas wells. Furthermore, gas turbines and supercritical CO_2_ power generation systems can be deployed directly at the well site to generate electricity from shale gas, thereby reducing transportation costs and enhancing overall efficiency.

**Figure 2:**
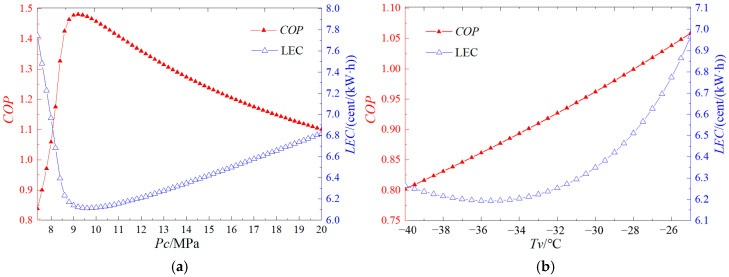
Effects on the T-CO_2_ refrigeration cycle performance parameters of the operating parameters (**a**) Cooling pressure, (**b**) Evaporating temperature.

To ensure the efficacy of shale gas production, it is imperative to maintain the temperature of the CO_2_ entering the formation at −20 °C, a condition that necessitates the implementation of a refrigeration cycle. Specifically, this refrigeration cycle employs a transcritical CO_2_ cycle. The influence of refrigeration cycle parameters on performance characteristics is illustrated in Figure 2. Figure 2a elucidates the impact of cooling pressure on the COP and levelized energy consumption (LEC) within the transcritical refrigeration cycle. As shown in Figure 2a, COP initially increases before decreasing as the condensing pressure rises, peaking at a condensing pressure of 9.2 MPa. Conversely, LEC exhibits an initial decrease followed by an increase, reaching its minimum at a condensing pressure of 9.6 MPa. This indicates the existence of an optimal condensing pressure for both COP and LEC to achieve their respective optimal values. Figure 2b delineates the effect of evaporation temperature on COP and LEC. According to Figure 2b, COP consistently increases with rising evaporation temperature, whereas LEC initially decreases and subsequently increases, attaining its minimum value at an evaporation temperature of −35.5 °C. Thus, while an increase in evaporation temperature benefits COP, it adversely affects LEC. This phenomenon can be attributed to the fact that, with a fixed cooling temperature, an increase in evaporation temperature reduces the temperature difference across the evaporator, leading to a required increase in the heat transfer area, which ultimately results in higher costs.

**Figure 3:**
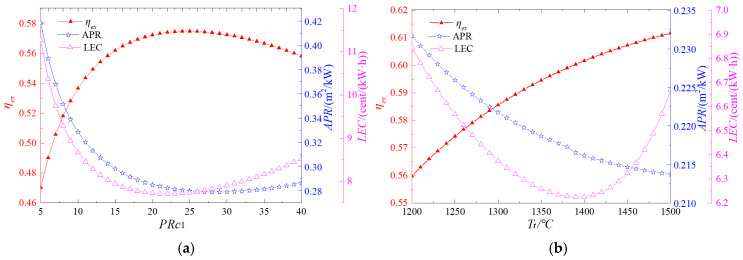
Effects of gas turbine operating parameters on system performance (**a**) Pressure ratio, (**b**) Inlet temperature of turbine.

Figure 3 illustrates the influence of the gas turbine pressure ratio and turbine inlet temperature on system performance. As shown in Figure 3a, the exergy efficiency initially increases and then decreases with a rise in the gas turbine pressure ratio, peaking at 57.5% at a pressure ratio of 25. Both the area per unit power output (APR) and levelized energy cost (LEC) exhibit a similar trend, decreasing initially and then increasing, with their minimum values occurring at pressure ratios of 29 and 22, respectively. These findings indicate that a moderate pressure ratio optimizes overall system performance. Figure 3b demonstrates that exergy efficiency improves with increasing turbine inlet temperature. This improvement is attributed to the higher specific enthalpy at the turbine inlet, which enhances power output and, consequently, exergy efficiency. However, as the turbine inlet temperature rises, the APR decreases, while the LEC initially declines before increasing, reaching its lowest value of 6.22 cents/(kW·h) at a turbine inlet temperature of 1390 °C. This behavior arises because higher turbine inlet temperatures boost power output but also increase operational costs. Therefore, an optimal turbine inlet temperature exists that minimizes the system cost rate.

**Figure 4:**
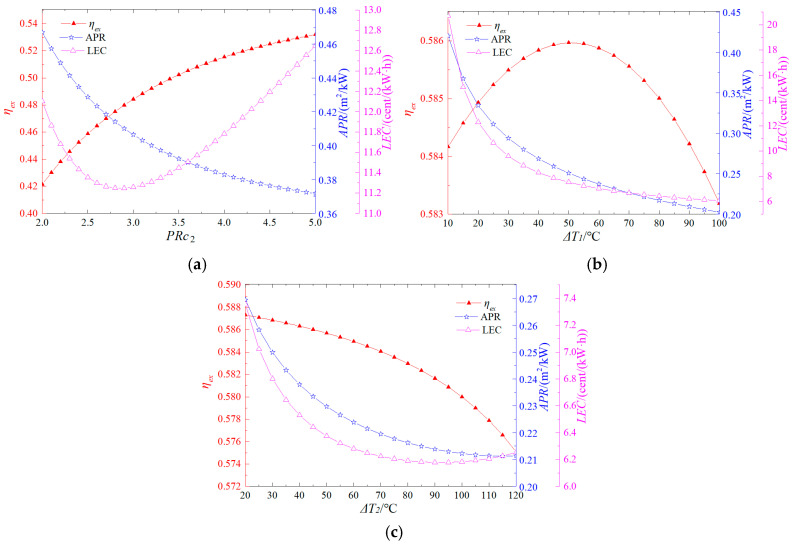
Effects of supercritical CO_2_ cycle operating parameters on system performance (**a**) Pressure ratio, (**b**) Temperature difference between the flue gas and the top cycle, (**c**) Temperature difference between the flue gas and the bottom cycle.

Figure 4 demonstrates the effects of the S-CO_2_ cycle’s pressure ratio, the temperature differences between the flue gas and both the top and bottom cycles on the system’s performance. As shown in Figure 4a, the system exergy efficiency increases monotonically with a rise in the supercritical CO_2_ cycle pressure ratio, while the area per unit power output (APR) exhibits a decreasing trend. The levelized cost of electricity (LCE) initially decreases and then increases as the pressure ratio rises, reaching its minimum value at a pressure ratio of 2.9. This behavior is due to the higher output power achieved at increased pressure ratios, which, however, also result in higher capital expenditures. Figure 4b demonstrates that the system exergy efficiency increases initially and then decreases as the temperature difference between the flue gas and the top cycle rises, peaking at 58.6% when the temperature difference is 50 °C. Meanwhile, both the APR and LCE consistently decline with increasing temperature differences. This trend occurs because larger temperature differences reduce output power, which in turn decreases the required heat exchange area, thereby lowering the cost of the heat exchanger. Figure 4c reveals that the system exergy efficiency continuously decreases as the temperature difference between the flue gas and the bottom cycle increases. Both the APR and LCE initially decrease before increasing, achieving their minimum values at temperature differences of 115 °C and 90 °C, respectively. This phenomenon arises because, at first, the reduction in the heat exchanger’s cost due to a smaller required heat exchange area dominates. However, as the temperature difference continues to grow, the corresponding decline in output power becomes significant, ultimately exerting a stronger influence on system efficiency and economics.

**Figure 5:**
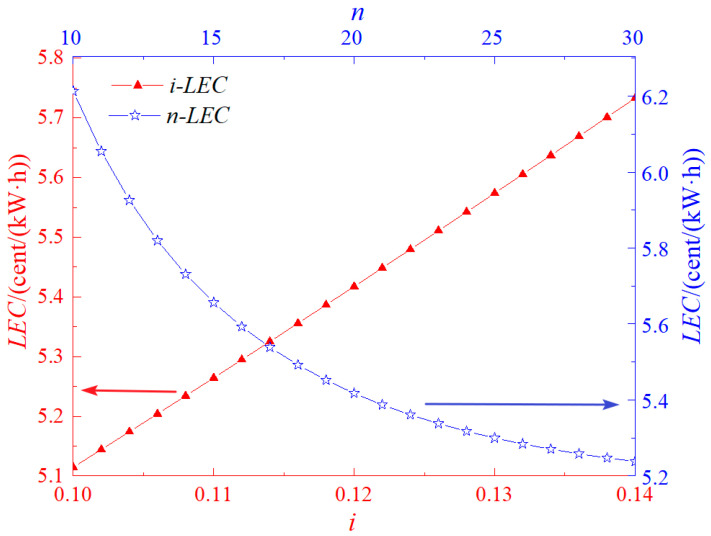
Effects of *i* and *n* on LEC.

Figure 5 presents the influence patterns of the interest rate (*i*) and the system’s service life (*n*) on *LEC*. As depicted in Figure 5, *LEC* rises with the increase in the interest rate, while it drops as the service life extends. This pattern also validates the accuracy of the economic model.

### 4.2. Multi-Objective Optimization Study

As discussed in Section 4.1, the optimal values of each objective function correspond to different system operating parameters, demonstrating that no single set of parameters can simultaneously optimize all performance metrics. Moreover, a trade-off relationship exists among these performance metrics. To enhance the application prospects of the integrated shale gas development and utilization system, a multi-objective optimization approach has been adopted. In this approach, the various performance metrics are treated as objective functions, while the critical operating parameters serve as decision variables. Using multi-objective optimization methods, the optimal operating parameters can be identified. For problems involving m objective functions and n decision variables, the optimization function is defined as:

(40) $$\min F(X)={\left[f_{1}(X),f_{2}(X),f_{3}(X)\ldots f_{m}(X)\right]}^{T}$$

Subject to

$$\begin{array}{l} g_{j}(X)\leq 0,j=1,\ldots ,J\\ h_{k}(X)=0,k=1,\ldots ,K\\ x_{i,\min}\leq x_{i}\leq x_{i,\max},\;i=1,\ldots ,n \end{array}$$

where F(X) is the objective function vector; X is the decision variable vector; *g_j_*(*X*) and *h_k_*(*X*) are inequality constraints and equality constraints respectively. *x_i, min_*, and *x_i, max_* represent the upper and lower bounds of the decision variable, respectively.

Based on the findings from the parametric analysis, the operating parameters that significantly influence the performance of the shale gas development and utilization system were selected as decision variables. These parameters include the compressor pressure ratio of the gas turbine (*PRc*_1_), the inlet temperature of the gas turbine (*T*ₜ), the pressure ratio of the supercritical CO_2_ cycle (*PRc*_2_), the temperature difference between the flue gas and the supercritical CO_2_ in the top cycle (Δ*T*_1_), and the temperature difference between the flue gas and the supercritical CO_2_ in the bottom cycle (Δ*T*_2_). The upper and lower bounds for these decision variables are summarized in Table 3. The gas turbine blades utilize single-crystal superalloys as the substrate. Inside the blades, ribbed serpentine channels are designed for convective and impingement cooling. The surface is coated with thermal barrier coatings, and film cooling holes with advanced hole patterns are drilled in critical regions such as the leading edge and the suction surface. Air is employed as the cooling medium. These technologies, taken together, constitute a robust thermal protection system. Currently, gas turbines corresponding to *T*ₜ are commercially available, and the temperature range corresponds to E-class, F-class and H-class gas turbines.

**Figure 6:**
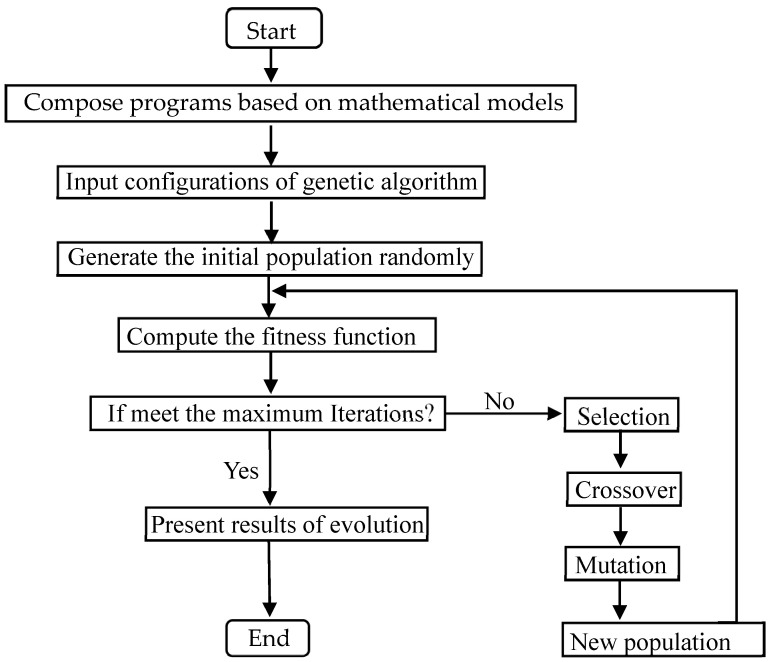
Flow chart of the simulation procedure.

To optimize the shale gas development and utilization system, a genetic algorithm was selected to perform multi-objective optimization, given the complexity of decision variables and the need for high optimization accuracy. The genetic algorithm is well-suited for handling such problems due to its robustness in exploring large solution spaces and balancing multiple objectives. Figure 6 depicts the flow chart of the simulation procedure. Table 4 outlines the key input parameters of the genetic algorithm used in this study.

After employing a genetic algorithm for multi-objective optimization, the resulting optimal solution set, known as the Pareto front, is obtained. All points within this solution set represent feasible solutions. To identify the most desirable optimal solution for operational parameters, the Technique for Order Preference by Similarity to an Ideal Solution (TOPSIS) method was selected. TOPSIS is a widely used approach for approximating the ideal solution in optimization problems.

The procedure involves the following steps: first, the matrix of feasible solutions is established and normalized to differentiate between ideal and non-ideal solutions. Then, the distances of each feasible solution from both the ideal and non-ideal solutions are calculated. Finally, a ranking analysis is performed to determine the optimal solution based on its proximity to the ideal solution.

#### 4.2.1. Double Objective Function Optimization

Double-objective optimization involves simultaneously optimizing and analyzing two objective functions to achieve an optimal equilibrium solution. For the system under study, which has three performance parameters-exergy efficiency (*η*ₓ), levelized energy cost (LEC), and area per unit power output (APR)-two objective functions can be selected based on specific application requirements. In this case, exergy efficiency and LEC were chosen for dual-objective optimization to improve system performance. Figure 5 illustrates the Pareto frontier solution of the optimized system, displaying all trade-off solutions between exergy efficiency and LEC. Decision-makers can select the best operating parameters according to their priorities.

**Figure 7:**
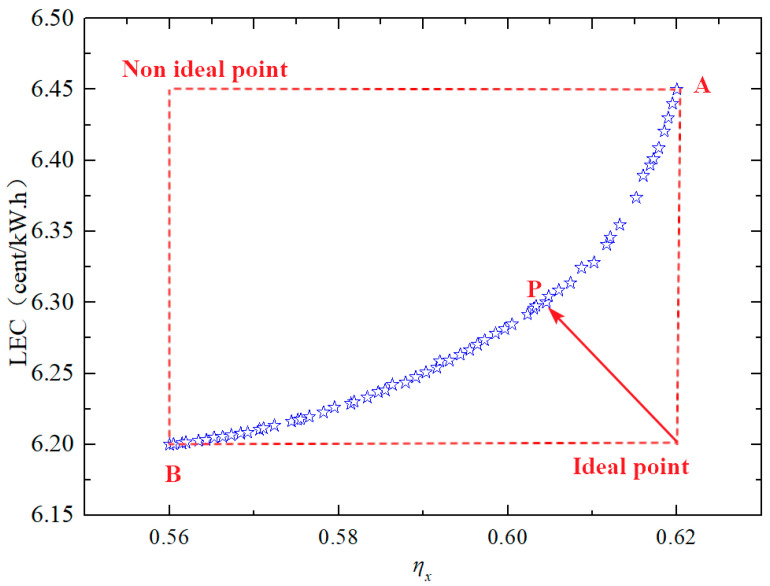
Pareto frontier of total product unit cost with exergy efficiency.

As shown in Figure 7, point A achieves an exergy efficiency of 62% and an LEC of 6.45 cents/(kW·h), indicating the best thermodynamic performance but the worst economic performance. Conversely, point B yields the lowest exergy efficiency (56%) and LEC (6.2 cents/(kW·h)), signifying the best economic performance but the poorest thermodynamic performance. Notably, no single point on the Pareto frontier can simultaneously maximize exergy efficiency and minimize the unit power cost.

In practical engineering, a balanced solution is required. While an ideal point that achieves both maximum exergy efficiency and minimum LEC does not exist, it serves as a reference for decision-making. Point P, which is closest to the ideal point, emerges as the optimal choice. The system at point P achieves an exergy efficiency of 60.5% and an LEC of 6.3 cents/(kW·h). The corresponding optimal operating parameters are: *PRc*_1_ = 28, *T*ₜ = 1380 °C, *PRc*_2_ = 3.2, Δ*T*_1_ = 73 °C, and Δ*T*_2_ = 62 °C.

#### 4.2.2. Three Objective Function Optimization

**Figure 8:**
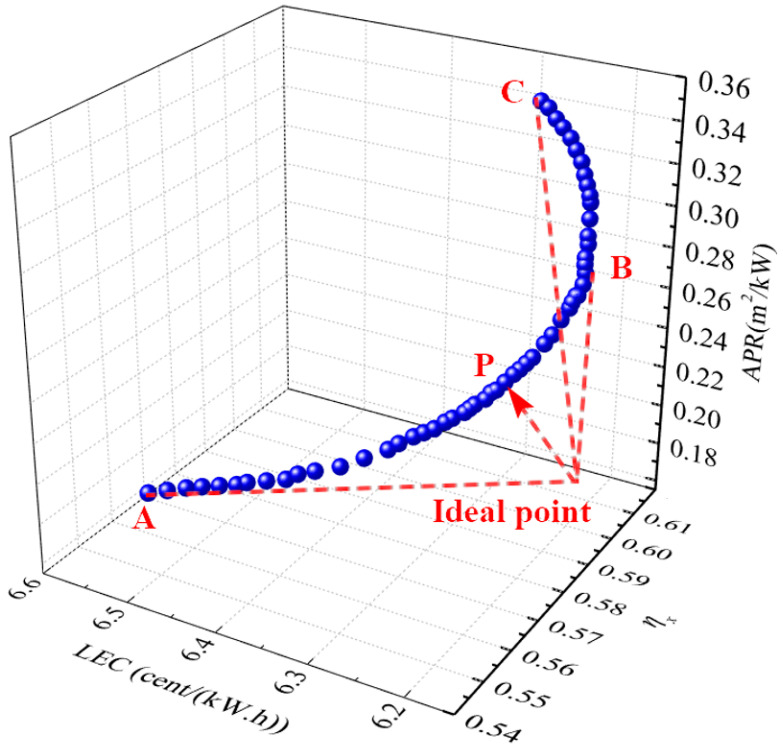
Three-dimensional optimization results for the system.

Exergy efficiency (*η*ₓ), LEC, and APR are considered as objective functions in this section to comprehensively evaluate the thermodynamic performance, economic performance, and spatial compactness of the combined cycle. The independent variables remain consistent with those in the previous section. The Pareto frontier solutions for system performance, obtained through optimization, are presented in Figure 8. These solutions represent all feasible, optimized outcomes, enabling decision-makers to select the best operating parameters based on specific needs. As illustrated in Figure 8, point C achieves the highest exergy efficiency of 61% and the lowest LEC of 6.194 cents/(kW·h), while point A exhibits the lowest APR of 0.2 m^2^/kW. This indicates that point C has the best thermodynamic performance, point B excels in economic performance, and point A performs best in spatial compactness. However, no single point satisfies all three-performance metrics simultaneously.

To address this trade-off, an ideal point—representing the highest exergy efficiency, lowest LEC, and smallest APR—is used as a reference in the decision-making process. Although this ideal point does not exist, the calculation identifies the point on the Pareto frontier nearest to it. This point, labeled as point P, is considered the optimal solution. The resulting system at point P achieves an exergy efficiency of 59.5%, an APR of 0.21 m^2^/kW, and an LEC of 6.3 cents/(kW·h). The corresponding optimal parameters are: *PRc*_1_ = 31, *T*ₜ = 1405 °C, *PRc*_2_ = 3.3, Δ*T*_1_ = 76 °C, and Δ*T*_2_ = 65 °C.

## 5. Conclusions

This paper presents an innovative and comprehensive supercritical CO_2_ system for shale gas development, comprising a supercritical CO_2_ shale gas extraction system, a gas turbine system, a supercritical CO_2_ power generation system, and a transcritical CO_2_ refrigeration system. CO_2_ is utilized as the working medium due to its significant environmental benefits. Through detailed thermodynamic, economic, and spatial compactness analyses, combined with multi-objective optimization of the integrated energy system, the following key conclusions were drawn:

(1) The parametric analysis revealed that there exists an optimal condensation pressure that both maximizes the coefficient of performance (COP) and minimizes levelized energy cost (LEC). Additionally, while an increase in evaporation temperature improves the COP, it does not positively impact the LEC.

(2) Key parameters, including the gas turbine pressure ratio (PRc_1_), turbine inlet temperature (Tₜ), supercritical CO_2_ pressure ratio (PRc_2_), and the temperature differences ΔT_1_ and ΔT_2_, significantly influence system performance. However, it is not feasible to simultaneously optimize all parameters to achieve the best overall system performance.

(3) Using exergy efficiency and LEC as objective functions, the optimization results showed an optimal exergy efficiency of 60.5% and an LEC of 6.3 cents/(kW·h). The corresponding optimal parameters were PRc_1_ = 28, Tₜ = 1380 °C, PRc_2_ = 3.2, ΔT_1_ = 73 °C, and ΔT_2_ = 62 °C.

(4) When optimizing for exergy efficiency, APR, and LEC, the system achieved an optimal exergy efficiency of 59.5%, an APR of 0.21 m^2^/kW, and an LEC of 6.3 cents/(kW·h). The corresponding optimal parameters were PRc_1_ = 31, Tₜ = 1405 °C, PRc_2_ = 3.3, ΔT_1_ = 76 °C, and ΔT_2_ = 65 °C.

## Figures and Tables

**Table 1:** Model Validation Using Data from Reference [25].

Operation and Performance Parameters		Reference	This Model
Input parameters	Compressor inlet temperature (°C)	32	32
	Turbine inlet pressure (MPa)	25	25
	Compressor inlet pressure (MPa)	7.63	7.63
	Isentropic efficiency of the turbine	85	85
	Isentropic efficiency of the compressor	80	80
Output parameters	Exergy efficiency (%)	42.68	44.15
	Specific investment cost ($/kW)	2681.7	2593.1

**Table 2:** Model Validation Using Data from Reference [26].

Operation and Performance Parameters		Reference	This Model
Input parameters	Outlet temperature of the cooler (°C)	32	32
	Isentropic efficiency of the compressor	80	80
	Pinch point temperature difference (K)	5	5
	Evaporation temperature (°C)	−15	−15
Output parameters	Cooling capacity (kW)	46.86	48.03
	COP	2.33	2.39
	Exergy efficiency (%)	10.64	10.96

**Table 3:** Upper and lower boundaries of the decision variable.

Decision Variables	Lower Bound	Upper Bound
*PRc_1_*	5	40
*T_t_*/°C	1200	1500
*PRc_2_*	2	6
Δ*T_1_*/°C	20	100
Δ*T_2_*/°C	20	100

**Table 4:** The input parameters of genetic algorithm.

Parameters	Values
Crossover fraction	0.8
Migration fraction	0.2
Pareto front population fraction	0.6
Generations size	100
Function tolerance	10^−4^
Constraint tolerance	10^−6^

## Data Availability

Data is contained within the article. The original contributions presented in this study are included in the article. Further inquiries can be directed to the corresponding author.

## Nomenclature

Glossary		Subscripts	
*A*	heat transfer area, m^2^	0	ambient (temperature)
*C*	compressor	1,2	state points
*CC*	combustion chamber	*k*	the *kth* component
*T*1	turbine	*h*	heat fluid
*T*2	gas turbine	*c*	cold fluid
*R*	storage tank	*i*	inlet
*P*	pump	*o*	outlet
*H*	heat exchanger	*v*	evaporation
*h*	specific enthalpy, kJ/kg	*H*	heat exchanger
*s*	specific entropy, kJ/(kg K)	*D*	destruction
S-CO_2_	supercritical CO_2_ cycle	*C*1	compressor 1
T-CO_2_	transcritical CO_2_ refrigeration cycle	*C*1	compressor 1
*m*	mass flow rate, kg/s	*W*	work
*c*	specific heat capacity, kJ/(kg K)	*Q*	heat
*P*	pressure, bar	*ex*	exergy
*Q*	thermal power, kW	*net*	net power
*T*	temperature, ℃	Greek symbols	
*U*	heat transfer coefficient, kW/m^2^ K	*η_c_*	isentropic efficiency of compressor
*W*	power output, kW	*η_t_*	isentropic efficiency of turbine
*W_c_*	compressor power, kW	*η_th_*	thermal efficiency
*E*	exergy, kW	*η_ex_*	exergy efficiency

## References

[1] Ou C., You Z. (2024). Review of CO_2_ utilization and storage in adsorption-type unconventional natural gas reservoirs. Fuel.

[2] Zhao X., Chen Z., Wang B., Liao X., Li D., Zhou B. (2023). A Multi-medium and Multi-mechanism model for CO_2_ injection and storage in fractured shale gas reservoirs. Fuel.

[3] Hu H., Zhu Y.Q., Li S.Y., Li Z. (2021). Effects of green energy development on population growth and employment: Evidence from shale gas exploitation in Chongqing, China. Pet. Sci..

[4] Gao S., Guan Q., Dong D., Huang F. (2021). Environmental risks of shale gas exploitation and solutions for clean shale gas production in China. Front. Earth Sci..

[5] Wu F., Zhou Z., Zhang S., Cheng F., Tong Y., Li L., Zhang B., Zeng X., Li H., Wang D. (2023). Toxicity identification evaluation for hydraulic fracturing flowback and produced water during shale gas exploitation in China: Evidence from tissue residues and gene expression. Water Res..

[6] Irfan M., Gohar F., Jing Y. (2023). Shale Gas Exploitation and Utilization. IJLAI Trans. Sci. Eng..

[7] Bellani J., Verma H.K., Khatri D., Makwana D., Shah M. (2021). Shale gas: A step toward sustainable energy future. J. Pet. Explor. Prod. Technol..

[8] Guo J., Lu Q., He Y. (2023). Key issues and explorations in shale gas fracturing. Nat. Gas Ind. B.

[9] Xie W., Chen S., Wang M., Yu Z., Wang H. (2021). Progress and prospects of supercritical CO_2_ application in the exploitation of shale gas reservoirs. Energy Fuels.

[10] Shen W., Ma T., Zuo L., Yang X., Cai J. (2024). Advances and Prospects of Supercritical CO_2_ for Shale Gas Extraction and Geological Sequestration in Gas Shale Reservoirs. Energy Fuels.

[11] Lyu Q., Tan J., Li L., Ju Y., Busch A., Wood D.A., Ranjith P.G., Middleton R., Shu B., Hu C. (2021). The role of supercritical carbon dioxide for recovery of shale gas and sequestration in gas shale reservoirs. Energy Environ. Sci..

[12] Kuang N., Zhou J., Tian S., Dong Z., Zhang J., Wang H., Chen Y., Zhong Y. (2024). Fluid–Solid Coupling Model with the Multiple Flow Mechanism for CO_2_-Enhanced Shale Gas Recovery and CO_2_ Sequestration. Energy Fuels.

[13] Shen Z., Wang H., Li G. (2010). Feasibility analysis of coiled tubing drilling with supercritical carbon dioxide. Pet. Explor. Dev..

[14] Akbari N. (2018). Introducing and 3E (energy, exergy, economic) analysis of an integrated transcritical CO_2_ Rankine cycle, Stirling power cycle and LNG regasification process. Appl. Therm. Eng..

[15] Zou L., Liu Y., Yu J. (2025). Thermodynamic and environment analysis of a modified transcritical CO_2_ refrigeration cycle integrated with ejector and subcooler. Int. J. Refrig..

[16] Aredokou L.O., Chegnimonhan V.K., Guidi C.T., Kapen P.T., Kounouhewa B. (2024). Transcritical carbon dioxide refrigeration and air conditioning cycles and applications: State of the art. Res. J. Eng. Sci..

[17] Liu Y., Zhao Y., Yang Q., Liu G., Li L. (2024). Research on compression process and compressors in supercritical carbon dioxide power cycle systems: A review. Energy.

[18] Cao Y., Zhan J., Cao Q., Si F. (2022). Techno-economic analysis of cascaded supercritical carbon dioxide combined cycles for exhaust heat recovery of typical gas turbines. Energy Convers. Manag..

[19] Chen J., Liu L., Liao G., Zhang F., Tan S. (2023). Design and off-design performance analysis of supercritical carbon dioxide Brayton cycles for gas turbine waste heat recovery. Appl. Therm. Eng..

[20] Siddiqui M.E., Almitani K.H. (2020). Proposal and thermodynamic assessment of s-CO_2_ brayton cycle layout for improved heat recovery. Entropy.

[21] Siddiqui M.E., Almitani K.H. (2020). Energy and exergy assessment of s-CO_2_ Brayton cycle coupled with a solar tower system. Processes.

[22] Al-Sulaiman F.A., Atif M. (2015). Performance comparison of different supercritical carbon dioxide Brayton cycles integrated with a solar power tower. Energy.

[23] Nami H., Mahmoudi S.M.S., Nemati A. (2017). Exergy, economic and environmental impact assessment and optimization of a novel cogeneration system including a gas turbine, a supercritical CO_2_ and an organic Rankine cycle (GT-HRSG/S-CO_2_). Appl. Therm. Eng..

[24] Feng Y., Hung T.C., Greg K., Zhang Y., Li B., Yang J. (2015). Thermoeconomic comparison between pure and mixture working fluids of organic Rankine cycles (ORCs) for low temperature waste heat recovery. Energy Convers. Manag..

[25] Li B., Wang S.S., Wang K., Song L. (2021). Comparative investigation on the supercritical carbon dioxide power cycle for waste heat recovery of gas turbine. Energy Convers. Manag..

[26] Liang Y., Sun Z., Dong M., Lu J., Yu Z. (2020). Investigation of a refrigeration system based on combined supercritical CO_2_ power and transcritical CO_2_ refrigeration cycles by waste heat recovery of engine. Int. J. Refrig..

